# Heterotopic Triplet Pregnancy After Assisted Reproductive Techniques: A Systematic Review

**DOI:** 10.7759/cureus.75997

**Published:** 2024-12-19

**Authors:** Ioannis Korkontzelos, Petros Papalexis, Vasiliki E Georgakopoulou, Pantelina-Danai Korkontzelou, George Mpourazanis, Kalomoira Dosiou, Theodoros Kalampokas, Georgios Adonakis

**Affiliations:** 1 Department of Obstetrics and Gynecology, Ioannina State General Hospital “G. Chatzikosta”, Ioannina, GRC; 2 Unit of Endocrinology, First Department of Internal Medicine, Laiko General Hospital, Athens, GRC; 3 Department of Pathophysiology/Pulmonology, Laiko General Hospital, Athens, GRC; 4 Medical School, Medical University of Sofia, Sofia, BGR; 5 Department of Obstetrics and Gynecology, Aretaieion University Hospital, Athens, GRC; 6 Department of Obstetrics and Gynecology, School of Health Sciences, University of Patras, Patras, GRC

**Keywords:** assisted reproductive techniques, ectopic rupture, hemorrhage, heterotopic triplet pregnancy, in vitro fertilization

## Abstract

Heterotopic pregnancy is defined as the simultaneous presence of an intrauterine and an extrauterine pregnancy and is considered a rare condition. As a part of this entity, heterotopic triplet pregnancy, defined as the presence of three embryos, with at least one being ectopic, is exceedingly rare. In recent years, the broad use of assisted reproductive techniques to help infertile couples has contributed to the constant rise of non-spontaneous heterotopic triplets. An analysis of all the cases reported in the English literature could provide useful messages to clinicians. This literature review had the aim to provide an analytic overview of all the cases of heterotopic triplet pregnancy after ovulation induction and assisted reproductive techniques published in the English literature by searching the National Library of Medicine (PubMed), Scopus, and Google Scholar until December 2023. We identified 57 articles of heterotopic triplets after assisted reproductive techniques and recorded the year, maternal age, gravida/para, medical history, medication used and mode of conception, number of embryos and stage of cells transferred, number of embryos in utero and ectopic embryos, time of diagnosis after ovulation induction or embryo transfer, symptoms, rupture of the ectopic pregnancy with hemoperitoneum, complications in pregnancy, obstetric outcome, weeks of delivery, birth-weight status and sex of the infants. Although this situation could be life-threatening and should alert the medical community, misdiagnosis or delayed diagnosis is still present. The commonest site of ectopic is in the salpinx. Currently, there is no consensus on the treatment strategy for heterotopic triplets, and all the data is derived from case reports. It is important to highlight the current recommendation of transferring one, or at most two, fertilized embryos during a single ovarian-controlled cycle to minimize the risk of multiple gestations and associated complications. Clinicians must remain vigilant, as pregnancies following in vitro fertilization (IVF) are considered “high-risk.” Complications, such as multiple pregnancies, preterm delivery, or maternal-fetal health issues, may arise. Early diagnosis, appropriate surgical or conservative interventions, and rigorous follow-up are essential to ensure optimal maternal and fetal outcomes.

## Introduction and background

Ectopic pregnancy is defined as the presence of an embryo outside of the uterus. Heterotopic pregnancy (HP) is characterized by the simultaneous coexistence of an intrauterine and extrauterine pregnancy. In more complex cases, the uterus may implant one or more fetuses, while the remaining embryos may be present in different implantation sites [1, 2]. The word “heterotopic” originates from the Greek phrase “eteros topos,” which means sited in “another place” and in obstetrical terms, in a wrong place outside the uterus.

Heterotopic triplet pregnancy (HTP) predisposes the existence of three embryos and could present as a single or twin intrauterine gestation, with the rest of the embryos/embryo being ectopic. HTP is still considered exceptionally rare, especially in spontaneous pregnancies, but is increasing steadily in recent years with the increased use of assisted reproductive techniques (ART) in infertile couples [2]. In ascending order, the ectopic implantation site could be the cervix, the cornua, the salpinx, the ovary, and the abdomen. The literature has not yet mentioned any cases of heterotopic triplet abdominal pregnancy.

Until 1984, previous articles used the term "combined" ectopic or "combined" heterotopic pregnancy to describe the presence of three embryos [3,4]. The phrase “heterotopic triplet pregnancy” was first mentioned by Goldman in 1991 [5], and with the increased number of similar cases, it was actually established by most of the authors in the title of their manuscript, especially after 1998 [6-9].

The aim of this systematic review is to analyze all the reported cases of heterotopic triplet pregnancies following ovulation induction and ART published in the English literature. This review seeks to collect comprehensive data on factors such as maternal age, medical history, the type of ART used, and pregnancy outcomes. The goal is to raise awareness concerning the challenges of diagnosing and managing heterotopic triplet pregnancies, which are still rare but increasing steadily due to the growing use of ART, and also provide useful messages to clinicians.

## Review

Materials and methods

Defining the Research Question (PICO Framework)

We defined the research question through the PICO framework as follows: Population (P): patients diagnosed with heterotopic triplet pregnancies, particularly those who have undergone ART or related treatments; Intervention (I): the primary interventions are ART such as in vitro fertilization (IVF), ovulation induction, or embryo transfer; Comparison (C): case reports often do not include a comparison group, but potential comparisons could be with spontaneous pregnancies or different ART procedures; Outcome (O): outcomes will focus on pregnancy results, maternal complications (e.g., hemorrhage, ectopic rupture), and fetal outcomes (e.g., live birth, premature delivery, neonatal health). Table 1 illustrates the PICO framework.

**Table 1:** PICO framework.

PICOS Element	Definition	Study-specific information
P-Population	The target group under investigation	Patients diagnosed with heterotopic triplet pregnancy, specifically those after ART.
I-Intervention	The primary treatment or procedure applied	Assisted reproductive techniques (ART), including In vitro fertilization (IVF)-Ovulation induction-Embryo transfer.
C-Comparison	A group or standard for comparison (if applicable)	Not explicitly applicable; comparison is implied between spontaneous vs. ART-conceived pregnancies or alternative ART protocols.
O-Outcomes	Key results or consequences	1. Maternal complications: hemorrhage, hemoperitoneum, ectopic rupture. 2. Obstetric outcomes: -Pregnancy viability, fetal survival, delivery mode, preterm birth, neonatal health.

Search Strategy

For comprehensive coverage, a systematic search was conducted using the databases PubMed, EMBASE, Scopus, Google Scholar, and Cochrane Library. A combination of Medical Subject Headings (MeSH) terms and keywords were employed, with Boolean operators (AND, OR) used to refine and combine relevant concepts. The search terms were structured around the population, intervention, and outcomes. Population-related search terms included “Heterotopic pregnancy”, “Triplet pregnancy”, “Ectopic pregnancy”, “Multiple pregnancy”, and the MeSH term “Pregnancy, Heterotopic”. Intervention-related terms encompassed “Assisted reproductive techniques”, “Ovulation induction”, “In vitro fertilization (IVF)”, and “Embryo transfer”. Outcome-related terms included “Pregnancy outcome”, “Maternal complications”, “Obstetric outcomes”, and “Fetal outcome”. Additionally, the search strategy incorporated terms specific to case reports, such as “case report” and “case series”, applying relevant filters where available. For instance, in PubMed, the search strategy combined population, intervention, and outcome terms as follows: (“Heterotopic pregnancy” OR “Ectopic pregnancy” OR “Triplet pregnancy” OR “Multiple pregnancy”) AND (“Assisted reproductive techniques” OR “In vitro fertilization” OR “IVF” OR “Embryo transfer”) AND (“Pregnancy outcome” OR “Maternal complications” OR “Obstetric outcomes” OR “Fetal outcome”) AND (“case report” OR “case series”). This systematic approach ensured the comprehensive identification of relevant case reports and clinical outcomes related to heterotopic and multiple pregnancies in the context of assisted reproductive technologies.

Inclusion/Exclusion Criteria

The inclusion and exclusion criteria for this review were clearly defined to ensure the selection of relevant studies. The inclusion criteria comprised case reports published in English, focusing on heterotopic triplet pregnancies following ART or IVF, and articles published within a specified date range. The exclusion criteria involved studies that were not case reports, such as reviews and clinical trials, as well as those that did not specifically address heterotopic triplet pregnancies. Additionally, editorials, opinion pieces, and other non-clinical articles were excluded. These criteria were applied to maintain a focused analysis on clinically relevant case reports related to heterotopic triplet pregnancies in the context of ART or IVF.

PRISMA Process

The PRISMA process for this systematic review on heterotopic triplet pregnancy after ART involved the following steps:

Identification: A total of 150 studies were identified, and the search was limited to case reports and clinical studies published in English up to December 2023.

Screening: After removing 30 duplicates, the titles and abstracts of 120 studies were reviewed based on predefined inclusion and exclusion criteria. Studies were included if they focused on heterotopic triplet pregnancies following ART, were published in peer-reviewed journals, and reported patient outcomes. Exclusion criteria resulted in the removal of 40 studies, which included reviews, editorials, clinical trials without a focus on heterotopic triplet pregnancies, and studies not in English.

Eligibility: Full-text articles for 80 studies were assessed for eligibility. During this phase, studies that did not specifically address heterotopic triplet pregnancies in ART cases or lacked clinical relevance were excluded. This assessment led to the exclusion of 23 studies, leaving 57 studies for further evaluation.

**Figure 1:**
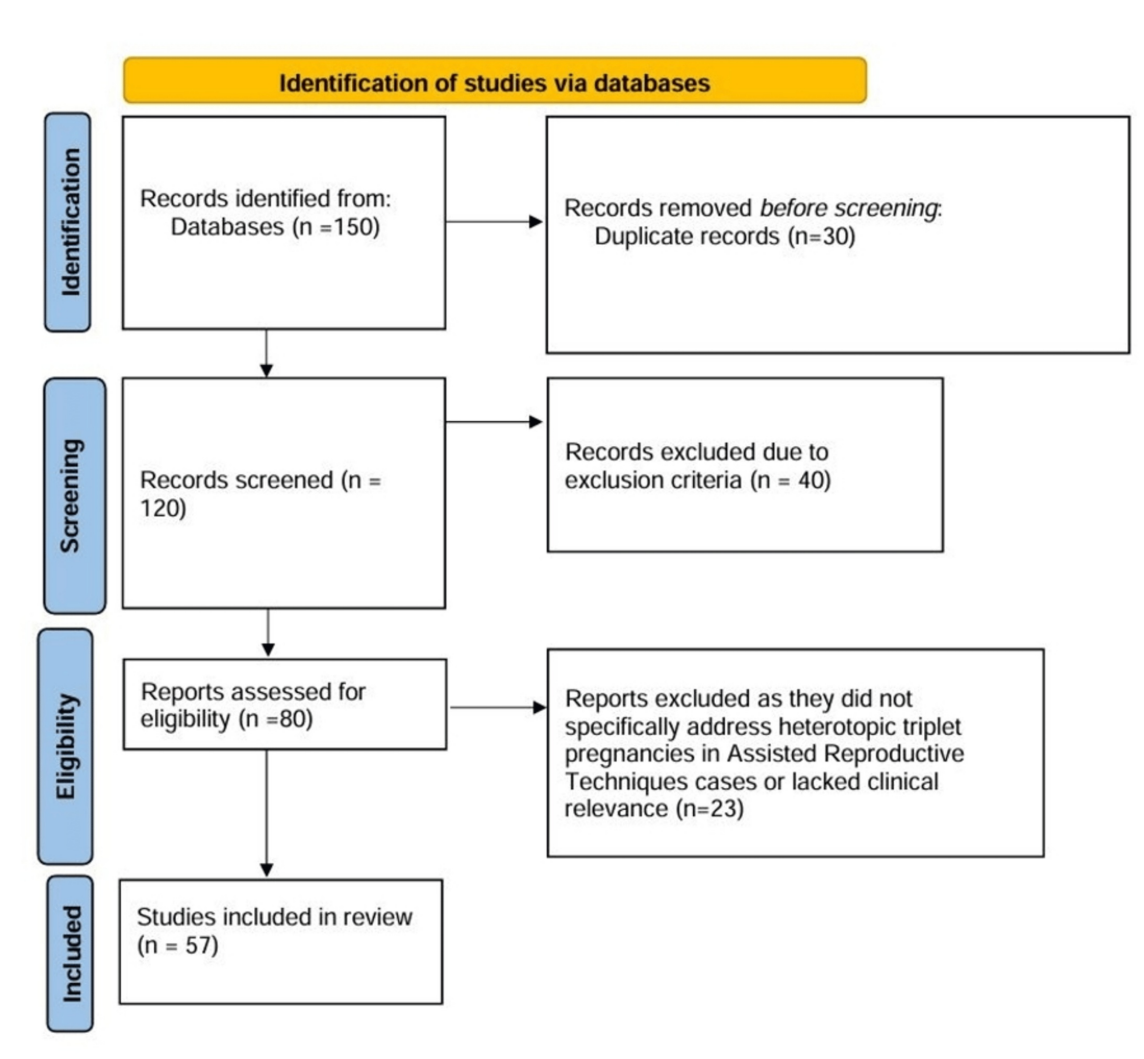
Flowdiagram of the study selection process.

Inclusion: Ultimately, 57 case reports and clinical studies met the inclusion criteria and were incorporated into the systematic review. A PRISMA flowchart was created to visually represent the number of studies at each stage of the process (Figure 1).

These 57 studies provided comprehensive data on heterotopic triplet pregnancies and associated ART interventions (Figure 1).

Quality Assessment

The quality assessment of the selected studies followed a structured framework to ensure the rigor and reliability of findings. Critical appraisal tools, such as the critical appraisal skills program (CASP) checklist for case reports, were used to evaluate essential elements, including patient demographics, ART intervention details, and outcomes. Methodological soundness, data completeness, and outcome relevance were key criteria. A Risk of Bias assessment categorized studies based on the potential for selection or reporting bias and conflicts of interest (Appendix A). Consistency and completeness of data, such as maternal age, ART procedures, and complications, further informed quality judgments, with missing data lowering the study’s rating. Lastly, outcome validity was scrutinized, emphasizing accurate diagnosis and management of heterotopic triplet pregnancies, ensuring the final review was based on high-quality, unbiased studies.

Data Extraction

The following data were extracted from the selected articles: a) Patient Demographics: Age, medical history, gravida/para status; b) Intervention Details: Type of ART (e.g., IVF, ovulation induction), medication used, number of embryos transferred, and stage of cells and c) Outcomes: Maternal outcomes (complications, surgical interventions), fetal outcomes (live birth, premature delivery, neonatal health).

Results

We identified 57 articles of heterotopic triplets after assisted reproductive techniques and recorded the year, maternal age, gravida/para, medical history, medication used and mode of conception, number of embryos and stage of cells transferred, number of embryos in utero and ectopic embryos, time of diagnosis after ovulation induction or embryo transfer, symptoms, rupture of the ectopic pregnancy with hemoperitoneum, complications in pregnancy, obstetric outcome, weeks of delivery, birth-weight status and sex of the infants.

The maternal age ranged from 24-year-old as the youngest to 47-year-old as the oldest, with a mean age of 32.36±4.84 years. From the medical history, we noted that the infertility etiology is multifactorial in most cases. The predominant infertility reason is the tubal factor, single (salpingectomy salpingostomy, tuboplasty, hydrosalpinx, ectopic, occlusion) or mostly combined with other causes, found in 23/57 (40.35%), [2-4, 8-27], followed by male infertility single or combined in 8/57 (14%), [18, 28-34]. Endometriosis is reported in 3/57 cases (5.26%) [27,35,36]. Diethyl Stilbestrol Syndrome was noted in 2/57 (3.50%) cases, which were reported mainly in the first cases [5,35]. There was a single case in 1/57 (1.75%) with pituitary adenoma [37], while in 6/57 (10.5%), the medical history and the cause/causes of infertility are not stated [6,38-42] (Table 2).

**Table 2:** Summary of the studies findings. G/P: gravida/para;  DES: diethylstilbestrol syndrome; PID: pelvic inflammatory disease; PCOS: polycystic ovary syndrome; Lap: laparoscopy; NS: not stated; IVF: in vitro fertilization; ET: embryo transfer; ICSI: intra cytoplasmatic sperm injection; FSH: follicle stimulating hormone; LH: luteinizing hormone; HCG: human chorionic gonadotropin; HMG: human menopausal gonadotropin; GnRH: gonadotropin releasing hormone; MESA: microepididymal sperm aspiration; MTX: methotrexate; KCL: potassium chloride; D/C: dilation/curettage; MC: monochorionic twins; DC: dichorionic twins; PROM: premature rupture of membranes; TPL: threatened preterm labor; OHSS: ovarian hyperstimulation syndrome; SCEP: cesarean scar ectopic pregnancy; F: female, M: male; C/S: cesarean section.

N	Authors	Year	Age	G/P	Medical history/infertility	Medication	Conception	No Embryos	Stage(cells)	US-(Sacs in utero)	Ectopic	Diagnosis	Symptoms	Treatment	Complications/pregnancy	Obstetric outcome	Delivery (weeks)	Birth weight/Status
1	Payne S et al. [43]	1971	27	G1 P1	Involuntary infertility	Prednisone 2.5mg/4dtp + Clomiphene 50mg (4^th^-8^th^)	Ovulation induction – spontaneous	NS	NS	2	Right cornual	48 days	Abdominal pain	Laparotomy-salpingectomy	Premature labor	CS	35	Twins-healthy 2400gr/2120gr
2	Walker TA et al. [44]	1972	33	-	Infertility	Clomiphene Citrate 50mg (4^th^-8^th^)	Ovulation induction – spontaneous	NS	NS	2	Right salpinx	8 weeks	Severe pain/pre-shock status	Laparotomy-salpingectomy/appendectomy	No	Vaginal delivery	37	Twins-healthy 2570gr/2260gr
3	Sondheimer SJ et al. [35]	1985	28	G0 P0	Primary infertility-endometriosis-DES (T-shaped uterus)	Gonadotropin	IVF-ET	5	5, 3, 2, 1	2	Left salpinx	41 days	Low abdominal pain	Lap-salpingectomy	Cerclage-premature labor	CS	33	NS
4	Yovich JL et al. [3]	1985	34	-	PID-bilateral hydrosalpinges-myomectomy	Clomiphene 100mg (2^th^-6^th^)+HMG+HCG	IVF-ET	5	4	2	Left salpix	8 weeks	Left pelvic pain	Lap/converted to laparotomy-salpingectomy	Demise 2^nd^ twin	Normal follow at 30 weeks	-	NS
5	Porter R et al. [4]	1986	33	G2 P2	Tubal factor	Clomiphene 100mg (3^th^-7^th^)+HMG 150IU (7-11)	IVF-ET	6	2,4	1	Right Twin tubal	6 weeks	Severe abdominal pain	Laparotomy-salpingectomy	No	Vaginal delivery	37	Healthy infant/3040gr
6	Hanf V et al. [10]	1990	30	G1	Tubal factor-bilateral hydrosalpinx	HMG+HCG	IVF-ET	4	4	1	Bilateral tubal	37 days	Severe abdominal pain-vaginal bleeding	Lap-salpingectomy bilateral	No	CS	38	Healthy
7	Goldman JA et al. [ 5]	1991	28	G1 P0	Diethyl stilbestrol syndrome	FSH+HMG	IVF-ET	4	4	1	Right twin tubal	6 weeks	Right pelvic pain/vaginal bleeding	Laparotomy	Premature labor	Vaginal delivery	30	Healthy infant/2600 gr
8	Chen SU et al. [11]	1992	32	G0 P0	Primary infertility/bilateral tuboplasty-fibrioplasty	FSH+HMG+HCG	IVF-ET	6	2 to 4	2	Right salpinx	10 weeks	Severe low abdominal pain	Laparotomy-repair of rupture	Abortion-D/C	-	-	-
9	Smith S et al. [12]	1993	27	G0 P0	Pelvic adhesions/bilateral hydrosalpinx	GnRH agonist+HMG+HCG	IVF-ET	4	Pronuclear	2	Right salpinx	32 days	Severe low abdominal pain	Laparotomy-removal	Premature labor	Premature labor/CS	24	Expired
10	Bassil S et al. [28]	1995	31	G0 P0	Male infertility	FSH+HMG+HCG	IVF-ET	3	NS	None	Right cornual triplets	35 days	No symptoms	Laparotomy-excision/horn reconstruction	Excision/corual pregnancy	-	-	-
11	Berliner I et al. [6]	1998	29	G1 P0	NS	75IU LH+FSH	Homologous intrauterine insemination	NS	NS	2	Left salpnix	5 weeks	Pelvic pain	Lap-salpingotomy	No	Vaginal delivery	37	Twins-healthy 2722gr/2863gr
12	Inion I et al. [7]	1998	30	G0 P2	PCOS/Ovulation Induction-Right ovarian torsion/laparotomy-2^nd^ ovarian torsion/laparoscopy	NS	IVF-Intrauterine insemination (donor)	2	8	2	Right adnexa	47 days	Right abdominal pain	Laparotomy-adnexectomy	Demise 2^nd^ twin	Vaginal delivery	NS	Healthy
13	Barnett A et al. [13]	1999	32	G0 P0	Primary infertility/tubal damage	NS	IVF	3	NS	2	Right salpinx	9 weeks	Right abdominal pain	Laparotomy-salpingectomy+appendectomy	No	CS	37	Twins-healthy 2080gr/2700gr
14	Nair P et al. [8]	1999	34	G2 P1	Lap left salpingostomy (hydrosalpinx)	GnRH analogs+gonadotropin	IVF/ET	3	NS	2	Left salpinx	34 days	Vaginal discharge/suprapubic tenderness	Laparotomy-left partial salpingectomy	No	CS	38	Twins-healthy
15	Ludwig M et al. [9]	1999	25	G0 P0	Primary infertility/tubal damage-PCOS/laparoscopy-ovarian abscess	HMG Long protocol	IVF-ET	3	NS	2	Right salpinx	48 days	Abdominal pain-OHSS	Lap-bilateral calpingectomy	No	NS	NS	NS
16	Oliveira FG et al. [29]	2002	31	G0 P0	Male factor-oligoasthenospermia	NS	IVF-ICSI	3	NS	2	Left Adnexa	38 days	Severe abdominal pain	Lap-salpingectomy	No	NS	38	Twins -healthy
17	Pan HS et al. [36]	2002	38	G3 P1	Endometriosis-pelvic adhesions-cervical stricture	HMG+FSH+HCG	IVF-ET(tubal due to cervical stenosis)	4	2,3,4	1	Bilateral tubal	5 weeks	Abdominal pain-vaginal bleeding	Laparotomy-bilateral salpingectomy	No	Vaginal	NS	Healthy Infant
18	Nikolaou DS et al. [14]	2002	31	G1 P0	PCOS/laparoscopic drilling/laparotomy-Left oophorectomy+partial salpingectomy	FSH+HCG	IVF-ET	2	NS	2	Left interstitial	28 days	Intermittent iliac fossa discomfort	Laparotomy-left tube incised and evacuated	Premature labor-MC twins	Vaginal	33	Twins –healthy 1400gr/1400gr
19	Hoopmann M. [30]	2003	39	G0 P0	Laparotomies (Crohn’s disease)-male Infertility	NS	IVF-ICSI	NS	NS	1	Bilateral tubal	6+6 weeks	No symptoms	Lap-bilateral linear salpingostomy	Empty sac syndrome	-	-	-
20	Sayin NC et al. [45]	2003	25	G3 P0	Infertility	Clomiphene citrate 100mg/day 5-9	Ovulation induction - Spontaneous	NS	NS	2	Left Salpinx	8 weeks	Acute abdomen	Laparotomy-left salpingectomy	Premature labor-MC twins	CS	35	Twins –healthy 2350gr/1900gr
21	Strelec M et al. [15]	2004	28	G2 P0	Infertility/tubal factor-Ectopic-right salpingectomy	HMG+HCG	IVF-ET	3	NS	2	NS	10+1 weeks	Severe pain-hypotensive/tachycardic	Lap-salpingectomy	PROM	CS	29	NS
22	Bhat SM et al. [16]	2004	31	G1 P1	Secondary infertility-Ectopic-left salpingostomy-right adnexectomy	GnRH antagonist+FSH+HCG	IVF-ET	4	NS	2	Right cornual	12 weeks	Abominal pain-collapsus	Laparotomy-repair of uterine cornual rupture	No	CS	36	Twins –healthy 2500gr/2800gr
23	Childs AJ et al. [38]	2005	30	G4 P2	NS	Gonadotropins	Uterine insemination	NS	NS	2	Left Salpinx	11 weeks	Abdominal pain	Lap-Salpingectomy	Missed abortion 2^nd^ twin (9weeks)	NS	NS	NS
24	Cirillo D et al. [39]	2006	28	G0 P0	NS	Gonadotropins	Natural conception	NS	NS	2	Right Ovary	8 weeks	Abdominal pain-vaginal bleeding/OHSS	Lap-Partial right ovarian resection	Premature labor	CS	32	Twins –healthy 1800gr/1950gr
25	Muller Vranjes A et al. [46]	2006	34	G0 P0	Infertility (5 years)/1 miscarriage/embolization for uterine myoma	Clomiphene citrate (2X50 mg), day 3-8	Not stated	NS	NS	2	Right Salpinx	10 weeks	Hemorrhagic shock	Lap-right salpingectomy	NS	NS	NS	NS
26	Dundar O et al. [31]	2006	35	G0P0	Male infertility	Clomiphene citrate	Uterine insemination	NS	NS	2	Right adnexa	10 weeks	Acute abdominal pain	Laparotomy-right salpingectomy	MC-monoamniotic twins	Follow up-16 weeks	-	-
27	Gupta A et al. [37]	2006	34	G0P0	Pituitary adenoma	2.5 mg Bromocriptine X3 per day	Spontaneous conception	NS	NS	1	Bilateral tubal	9 weeks	Shock	Laparotomy-left salpingectomy/right linear salpingotomy	Demise of intrauterine fetus/9 weeks/DC	-	-	-
28	Divry V et al. [17]	2007	32	G0 P0	Primary infertility (12 years)/tubal factor-bilateral salpingectomy	NS	IVF-ET	3	8	2	Right cornual	6 weeks	No symptoms	Lap/converted to laparotomy-excision (cornual)	NS	CS	31	Twins –healthy 1360gr/1590gr
29	Berkes E et al. [18]	2008	26	G1 P0	Secondary infertility/right salpingectomy-male factor (asthenoteratoazospermia)	GnRH agonist+HMG+1.25mg bromocryptine	IVF-ICSI	3	NS	0	Left isthmic+Twin tubal	33 days	Abdominal pain	MTX 50/100/100mg im-laparotomy/left interstitial salpingectomy	Empty uterus	-	-	-
30	Kasum M et al. [19]	2009	32	G0 P0	Primary infertility/distal occlusion of left tube-reconstructive surgery of the right tube/Left salpingectomy (ectopic)	NS	IVF-ET	NS	NS	1	Bilateral tubal	NS	Abdominal pain	Lap-left salpingectomy	-	Vaginal	40	Healthy infant
31	Bugatto F et al. [2]	2010	28	NS	Tubal factor (2 years)-Left tubal occlusion	Gonadotropin+HCG	IVF-ET	NS	NS	2	Left salpinx	12+4 weeks	Left iliac pain	Laparotomy/left salpingectomy	TPL (admission/cervical incompetence)	CS	31+2	Twins-healthy 1650gr/1800gr
32	Timor-Tritsch I et al [20]	2010	29	G5P0	Two miscarriages/Right ectopic-salpingectomy/bilateral ovarian ectopic-resection and left salpingectomy	NS	IVF-ET	2	NS	1	Right cornual MC Twin pregnancy	NS	NS-No symptoms (?)	Vaginal needle KCL injection/laparotomy-cornual resection	No	CS	37	NS
33	Tomic V et al. [21]	2011	31	NS	Primary infertility (tubal occlusion)	GnRH+r-FSH+HCG	IVF-ET	3	GI, GII	2	Right salpinx	7 weeks	Abdominal pain/OHSS	Lap/converted to laparotomy-right salpingectomy	No	CS	37	Twins-healthy 2450gr/2840gr
34	Litwicka K et al. [32]	2011	31	G1 P1	Secondary infertility/Male infertility (azoopsermia)/previous CS	GnRH antaonist+r-FSH	IVF-ICSI	3	NS	1	Two sacs - isthmic	6 weeks	No symptoms	Vaginal needle (20G) injection KCL(2ml)+MTX 15 mg	Contractions 28-34 weeks	Emergency CS/ hemorrhage	36	Miller syndrome
35	Okamura Y et al. [22]	2011	31	G2 P0	Infertility (6 years)/left pyosalpinx-right tubal obstruction-left salpingectomy	NS	IVF-ET	3	NS	2	Left tubal isthmic	8 weeks	Pre-shock status	Lap-left isthmic excision	Fetal demise 2^nd^ twin/8 weeks	Elective CS	37	Healthy infant-2828 gr
36	Bornstein E et al. [23]	2011	29	G5 P4	Lap right salpingectomy/2 miscarriages/2 bilateral ectopic ovarian pregnancies/Left salpingectomy	NS	IVF-ET	2	NS	1	Right cornual MC Twin pregnancy	8 weeks	No symptoms	Vaginal needle KCL-Bleeding-Laparotomy-Cornual evacuation/repair	No	Elective CS	37	NS
37	Gozukucuk M et al. [24]	2011	29	G1P0	Right tubal ectopic-salpingectomy/unexplained infertility	NS	IVF-ET	NS	NS	1	Twin cervical	NS	No symptoms	Dilatation and curettage	Empty sac syndrome	-	-	-
38	Osmanagaoglou M et al. [40]	2011	47	NS	NS	Leuprolide acetate 500 μg+225 IU r FSH+HCG	IVF-ET	3	NS	2	Left salpinx	9 weeks	Abdominal pain	Lap-Left salpingectomy	No	NS	35	Twins-healthy 2206/2426gr
39	Herrera E et al. [33]	2011	34	NS	Primary infertility/male actor-azoospermia	NS	IVF-ET (blastocyst transfer)	NS	NS	2	Left cornual	9 weeks	Abdominal pain	Laparotomy-Left salpingectomy	-	NS	NS	NS
40	Lin CK et al. [25]	2013	32	G2 P1	Secondary infertility (10 years)/tubal factor	200IU FSH+150IU HMG+HCG	MESA+ICSI	5	NS	2	Cervix	15 weeks	No	Sac needle aspitation+KCL 10ml KCL	Severe preeclampsia-Uterine atony/hysterectomy	CS-Hysterectomy	27	Twins DC-1000gr/705gr
41	Delrieu et al. [26]	2013	36	G3P3	Tubal ligation-unsuccessful tubal reanastomosis	GnRH agonist+HCG	IVF+ET	4	NS	1	Left salpinx+cervix	38 days	Abdominal pain	Lap-left salpingectomy/cervical mass excision	No	NS	NS	NS
42	Fukuda T et al. [47]	2014	32	G1 P0	History of ovulatory disturbance	GnRH agonist+225IU HMG+150IU r FSH+HCG	IVF-ET	3	Day 3	3	Cervix	8+3 weeks	No symptoms	Sac needle aspitation (19G)	Premature labor	Emergency CS	25	Twins DC-660gr/620gr
43	Felekis T et al. [1]	2014	32	G2 P0	Idiopathic infertility( 3years)	Clomiphene citrate (100mg for 5 days)+HCG	Uterine Insemination	NS	NS	1	Bilateral tubal	9 weeks	Abdominal pain/vaginal bleeding	Lap-left salpingectomy-right salpingostomy	No	Vaginal	39	Healthy infant
44	Buca DI et al. [48]	2015	37	G0 P0	Idiopathic infertility (5 years)	NS	IVF-ET	NS	NS	2	Right salpinx	9+4 weeks	Abdominal pain/nausea/vomiting	Laparotomy-Right salpingectomy	TPL (contractions/admission)	Elective CS	36+2	Twins –healthy 2460gr/2600gr
45	Kasap B et al. [49]	2015	32	G1P0	Unexplained infertility (4 years)	NS	ICSI-ET	3	GI	1	Bilateral tubal	21 days	No symptoms	Lap-Bilateral slapingectomy	Spontaneous abortion	-	-	-
46	Nitke S et al. [50]	2007	45	G0 P0	Primary infertility (5 years)	Donor eggs	IVF-ET	4	NS	1	2 sacs - cervix	7 weeks	No symptoms	Uterine arteries catheterized/84 mg MTX/Uterine embolization	Shrinkage of two cervical sacs/uterine sac	-	-	-
47	Hsieh BC et al. [51]	2004	38	G4 P2	Secondary infertility/ two CS	GnRH analogs+HCG	IVF-ET	3	NS	2	CSEP	21 days	No symptoms	Vaginal needle aspiration of the sac	Premature labor	NS	32	NS
48	Gaudier FL et al. [52]	1995	31	G0 P0	Primary infertility (7 years)	NS	IVF-ET	NS	NS	2	Right interstitial	15 weeks(MRI)	Acute abdominal pain	Laparotomy	Contractions-20 weeks	Premature labor/CS	34	Twins-healthy 2100gr/1500gr
49	Al Mulla A et al [53]	2018	38	-	Primary infertility-four unsuccessful ICSI	NS	IVF-ET	3	Blastocyst 3CC	2	Right salpinx	11 weeks	Schock	Lap-right salpingectomy	Premature labor	CS	34	Twins DC-healthy 2460gr/2600gr
50	Vitner D et al. [54]	2011	47	G0 P0	Egg donation	Controlled minimal ovarian stimulation	IVF-ICSI	NS	NS	2	Right salpinx	9+5 weeks	Severe abdominal pain	Laparotomy-right salpingectomy	No	-	Term	Twins DC- healthy
51	Mustafa KB et al. [55]	2016	37	G2 P0	Idiopathic infertility (6 years)	Gonadotropins+metformin+HCG	Natural conception	2	1,2	1	Bilateral tubal	8 weeks	Abdominal pain vaginal spotting/OHSS	Lap-bilateral salpingectomy	Spontaneous abortion	-	-	-
52	Jonler M et al. [56]	1995	31	G0 P0	Primary infertility (7 years)	NS	IVF-ET	NS	NS	1	Bilateral tubal	6+3 weeks	No symptoms	Lap-bilateral salpingotomy	Abortion-D/C	-	-	-
53	Riestenberg CK et al. [57]	2017	24	G0 P0	PCOS	NS	IVF-ET	NS	NS	1	Bilateral tubal	25 days	Abdominal pain- spotting	Lap-right salpingostomy/Left salpingectomy	Spontaneous abortion-D/C	-	-	-
54	Korkontzelos I et al. [34]	2019	38	G4 P0	Male factor (oligoasthenospermia)	NS	IVF-ICSI	3	4	2	Left salpinx	8+2 weeks	Abdominal pain/OHSS	Laparotomy-left salpingectomy	Demise 2^nd^ twin-TPL-cerclage	Elective CS	36+1	Healthy infant -1980gr
55	Bhattacharya R et al. [41]	2020	33	G2P0	NS	NS	IVF-ET	NS	NS	2	NS	11 weeks	Abdominal pain	Laparotomy-Left salpingectomy	DC twins-fetal demise	-	-	-
56	Morong J et al. [42]	2021	31	G3 P2	NS	Clomiphene Citrate 50 mg for 5 days	Natural conception	NS	NS	2	Left Salpinx	10+1 weeks-	Severe abdominal pain/vaginal bleeding	Lap-left salpingectomy	TPL	Elective CS	36+5	Twins-healthy
57	Bhoi NR et al. [27]	2023	35	G0P0	Hydrosalpinx-bilateral endometrioma-subserous fibroid	Goserelin acetate	IVF-ICSI	2	Grade 1	2	Left adnexa	7 weeks	No	Lap-cornual resection	No	Elective CS	36	Twins MC-healthy 2200gr/200gr

All the heterotopic pregnancies in the manuscript are presented after the use of ovulation induction drugs. In 18/57 (31.5%) cases the medication used is not stated. Clomiphene citrate in a dosage of 50 or 100 mg, single or in association with other drugs, was used in 9/57 (15.7%) [3,4,31,42-47], with natural conception occurring in 4/57 (7%) women [42-45]. In vitro fertilization (IVF) and embryo transfer (ET) was used in most of the cases, 34/57 (59.6%), while IVF plus intracytoplasmatic sperm injection (ICSI) was used in 8/57 (14%) cases, [27,29,30,32-34,48], including one patient with additional use of bromocriptine [18]. Intrauterine insemination, a less expensive method, was also used in a percentage of 6/57 (10.52%) cases [6,7,31,38,47,49] (Table 2).

The method of embryo transfer is not mentioned in most of the articles. From this study, we noticed that in the first early cases, the oocytes were transferred laparoscopically, while after 1993, in all the reported cases, the intervention was performed vaginally. It was also considered useful to state the number of embryos transferred and the stage of cells. Until 1993, the number of embryos transferred was four up to six. From 1993 until today, the number of embryos decreased between two and three, with only a few exceptions [16,25,33,36,50]. In 22/57 (38.6%) the number of embryos transferred is not stated (Table 2).

In cases where a single intrauterine pregnancy is noted, the distribution of ectopic twins was: twin bilateral tubal gestation in 11/57 (19.3%), twin pregnancy in a single tube in 2/57 (3.5%), and three corneal-isthmic twin pregnancies, 3/57 (5.2%). Cornual-interstitial-isthmic pregnancy with a single embryo was noted in 6/57 (10.5%), an empty uterus and cornual triplets in 1/57 (1.7%), one or more cervical embryos in 5/57 (8.7%), cesarean scar ectopic pregnancy in 1/57 (1.7%) and one ovarian ectopic pregnancy (1.7%). The right adnexal ectopic side is predominant and presented in 21/57 (36.8%), followed by the left side in 15/57 (26.3%) and bilateral tubal in 9/57 cases (15.7%). The earliest diagnosis was made after 21 days/3 weeks [51] and the latest in two patients at 15 weeks of gestation [33,52]. One of these two patients presented was referred for a suspected fetal anomaly, and the diagnosis of HTP was confirmed after a magnetic resonance imaging (MRI) (Table 2) [52].

A variety of symptoms was present, starting from intermittent iliac fossa discomfort. Abdominal pain, mild or severe (acute abdomen) with or without vaginal bleeding, was the main symptom in 41/57 (71.9%) patients, with the rest 16/57 (28%) being asymptomatic. Intra-abdominal hemoperitoneum was present in 30/57 (52.6%) patients (250 ml-2500 ml). In the remaining 25/57 (43.8%) of the cases, the ectopic was not ruptured, while in two cases, it was not stated. It has to be noted that, in 7/57 (12.28%) women, the admission was made in a pre-shock/hypotension-tachycardia or in a collapse/shock status (Table 2) [15,16,22,37,44,53].

Treatment options were mostly determined by the clinical status of the presenting patient, the site of the ectopic gestation, the existence of hemoperitoneum or bleeding, and the woman’s preferences. In total, 29/57 (50.8%) cases were treated by laparotomy, including three cases that started laparoscopically and were converted to laparotomy. Furthermore, two cases started with vaginal needle injection of potassium chloride (KCL) with or without methotrexate (MTX), [20, 40] and one case of initial intramuscular (im) injection of MTX which ended also in laparotomy [18]. In total initial treatment by vaginal approach (aspiration or injection of KCL) was performed in 6/57 (10.5%) patients [20,23,26,32,33,51]. Laparoscopically, they were treated in 22/57 (38.6%) cases. There was also a single case of 1/57 (1.7%) treated with dilation and curettage of an intrauterine and twin cervical pregnancy [54] and one case treated with an injection of MTX and uterine artery embolization [50]. As mentioned before, seven patients were admitted in a shock status. Furthermore, another eight patients were admitted with severe abdominal pain, as stated by the authors, reaching a total of 15/57 (26.3%) patients admitted in a serious condition. Nine out of these fifteen patients (60%) were treated by laparotomy, and interestingly, the rest, 6/15 (40%) by laparoscopy. There were also five cases where ovarian hyperstimulation syndrome (OHSS) was officially diagnosed with or without hemoperitoneum [9,21,34,39,55], and 3/5 (60%) of those patients were treated laparoscopically [9, 39], and 2/5 patients (40%) by laparotomy (Table 2) [21,34,55].

Complications in pregnancy and obstetric outcomes were recorded in most of the cases, 54/57 (94.7%). In 20/57 (35%), no symptoms occurred, and where stated, the patient had a successful outcome. Spontaneous abortion or empty sac syndrome was present in 9/57 (15.7%), followed by dilatation and curettage when necessary [11, 18, 30, 37, 48, 54-57]. Contractions threatened preterm labor, premature rupture of membranes, and premature labor (24-36 weeks) were noted in 13/57 (22.8%) women. One case suffered from severe preeclampsia at 27 weeks and ended with a cesarean section followed by uterine atony and hysterectomy (Table 2) [33].

Twenty-five women delivered by cesarean section, and nine vaginally. There were 5/57 (8.7%) twin intrauterine pregnancies with the fetal demise of the second twin [3,7,22,34,38], and three of them had a favorable outcome, while in two cases, the fetal demise of both dichorionic twins was noted. Four cases had very early premature labor (<29 weeks) [12, 15, 26, 33]. Out of these cases, one infant was delivered at 24 weeks and expired, two dichorionic twin cases were delivered at 27 and 25 weeks, and the last case was delivered at 29 weeks of gestation. In those last three cases, the neonatal status after birth and the follow-up is not mentioned. In 11/57 (19.3%) cases, the pregnancy ended in abortion or empty sac syndrome treated mostly by dilation and curettage, while in 18/57 (31.5%) cases, obstetrical follow-up and the outcome was not specified. Data also missed the sex of the newborns, but we recorded the birth of 19 female and 16 male infants. In total, out of all these 57 single or twin intrauterine gestations, 49 healthy infants were delivered, and one term neonate was diagnosed with Miller syndrome (Table 2) [32].

It was difficult to identify the surgical treatment modality that could be considered most appropriate and that also resulted in a successful obstetrical outcome. It has to be noted that many cases were complex, multifactorial, heterogeneous and received combined therapy intra-abdominal/operative, intra-cervical by needle injection and aspiration accompanied by conservative treatment with KCL and/or MTX. Thus, we decided to consider only cases that were delivered after 30 weeks and had a favorable outcome, while unreported neonatal status or severely complicated cases were excluded. Out of these 28 cases, nineteen (67.8%) were treated by laparotomy and nine (32.2%) by laparoscopy.

Discussion

Historically, the first reported naturally occurring heterotopic pregnancy was described by Duverney in 1708 after an autopsy of a patient who died from a ruptured ectopic pregnancy but simultaneously had an intrauterine pregnancy [58]. The first successful birth after in vitro fertilization process occurred in 1978 by Patric Steptoe and Robert Edwards [59]. However, their first attempt in 1976, where a human embryo was introduced into the uterus, ended up in an ectopic tubal pregnancy removed at 13 weeks of gestation. In 1971, Payne et al. [43] reported the first heterotopic triplet (twin intrauterine and right tubal) pregnancy in the English literature after ovulation induction with clomiphene citrate and corticosteroids. The first HTP resulting from in vitro fertilization and embryo transfer was reported by Sondheimer et al. [35]. 

Tal et al. [58] reviewed 139 cases of heterotopic pregnancies from 1971 up to 1993, independent of the number of embryos. Since then, HP has been increased but there was also a notable increase in “multiple”, or “combined” (triplet-quadruplet-quintuplet-sextuplet) heterotopic gestations. Concerning specifically heterotopic triplets, it is impressive that in a period of 21 years (1971-1992), only eight cases are recorded, while from 1993 until now, another forty-nine new cases of heterotopic triplets were added.

In spontaneous pregnancies, the incidence of HP is 1/30000. In pregnancies resulting from ART, the incidence is increasing dramatically from 1/100 up to 1/3600. Overall, the incidence of HP is estimated to be approximately 1/7000 to 1/15000 live births [1]. Patients treated with ovulation induction have a ratio of 33/10000, while after IVF, the risk is increased, approaching a ratio of 100/10000 [2]. This major difference in the statistics is attributed to the broad use of assisted reproductive techniques, basically ovarian stimulation, intrauterine insemination, transfer of many embryos in utero, the quality of the embryos, the hormonal milieu at the moment of transfer, transfer near the uterine horn, the amount of fluid used as media for the embryos, excessive pressure on the syringe and deep insertion of the catheter. Additional factors are considered: the frequent use of intrauterine devices (IUDs) and the higher incidence of pelvic inflammatory disease or endometriosis resulting in tubal damage or tubal malformation [1, 2].

Buggato et al. [2] published a review article, including also a case report of a twin intrauterine and a left ectopic pregnancy. To his knowledge, only 14 cases of HTPs, with two embryos being in utero and one extrauterine, were reported. In our review, we recorded in total 36/57 cases of a twin intrauterine and one ectopic fetus, reaching 63% and becoming the most common sub-category of HTP.

The diagnosis of HTP is challenging and difficult. Serial β-HCG levels are not helpful due to the presence of a concomitant intrauterine pregnancy. Transvaginal ultrasound (TVS) remains the preferred imaging method for diagnosing both tubal and non-tubal heterotopic pregnancies. Despite the widespread use of high-resolution vaginal sonography in clinical settings, its sensitivity for detecting heterotopic pregnancies remains low, as the condition is frequently missed or overlooked [14]. False assurance could be obtained by the ultrasonographic visualization of an intrauterine pregnancy, the absence of clinical symptoms, the presence of enlarged ovaries with multiple leutal cysts, or the presence of ovarian hyperstimulation syndrome with intra-abdominal free fluid.

In 1993, Fernandez et al. [60] stated that only 10% of all heterotopic pregnancies are diagnosed preoperatively. In heterotopic pregnancies, the most frequent implantation site is in the fallopian tube and most commonly in its ampullary segment. Kemp et al. [61] noticed that only 6-16% of ectopic tubal pregnancies show fetal heartbeat. However, increased vascularization within the structure visualized by color Doppler indicates ongoing ectopic pregnancy. Furthermore, a definite diagnosis could be made only when extrauterine fetal cardiac activity is seen on an ultrasound or in the operating theatre. Li et al., in a retrospective study, set certain sonographic criteria for the diagnosis of HP using TVS even if a visible intrauterine gestational sac is observed: (i) an inhomogenous adnexal mass, (ii) an empty extrauterine gestational sac seen as a hyperechoic ring, (iii) a yolk sac and/or a fetal pole with or without cardiac activity in an extra-uterine sac [62]. The sensitivity and specificity of TVS for the detection of HP in this study were reported to be 92.4% and 100%, respectively. In his relatively recent review, Bugatto et al. reported that in heterotopic pregnancies an accurate or suspected diagnosis was made only in 57% of the patients and most of them (60%) were diagnosed before 10 weeks of gestation, however a timely surgical treatment does not appear to improve the prognosis [2].

The diagnosis of HTP is becoming more complicated in cases of ruptured heterotopic pregnancies associated with ovarian hyperstimulation syndrome (OHSS). Both conditions can be linked to hypotension, tachycardia, abdominal pain, and the presence of free fluid in the abdominal cavity, along with a concurrent intrauterine pregnancy. A ruptured corpus luteum may also be misdiagnosed as HP, as both conditions can display the "ring of fire" sign on ultrasound. In contrast, women with OHSS typically present with normal hemoglobin levels, elevated hematocrit, and enlarged ovaries [14,21]. In our review, many cases mentioned enlarged ovaries with multiple luteal cysts, but only five cases of HTP accompanied by OHSS syndrome (9%) were officially recorded, and in those cases, late diagnosis occurred (Table 4).

The management of heterotopic triplet pregnancy focuses on removing the ectopic pregnancy while preserving the viable intrauterine embryo(s). Currently, there is no standardized treatment for HTP, and available data are based solely on case reports. Treatment strategies depend on factors such as gestational age at diagnosis, uterine size, the patient's clinical status, the location of the ectopic implantation, and the treating physician's expertise. Generally, heterotopic tubal pregnancy can be managed either conservatively or surgically. In cases of rupture or when the patient is hemodynamically unstable, laparotomy and removal of the ectopic sac is the traditionally preferred approach. Laparoscopy has also become the preferred method for final diagnostic confirmation in cases of dilemmas and has been increasingly used in the last years [29]. Laparoscopic injection of KCL or MTX into the tube has also been performed [63]. However, a review by Goldstein et al. [64] revealed that 55% of tubal heterotopic pregnancies treated with KCL injection required subsequent salpingectomy. Generally, salpingectomy is preferred in cases of uncontrolled bleeding or significant tubal damage. Salpingostomy, on the other hand, is indicated for stable patients with an unruptured ectopic sac smaller than 5 cm, especially if the contralateral tube is absent or damaged and the patient has a strong desire to preserve fertility [65, 66]. Αρχή φόρμαςDuring the operation, special attention should be provided for manipulations that will not disturb the uterus, leading to postoperative contractions also respecting the ovarian or collateral blood supply [2].

Conservative treatment requires early diagnosis and should be performed in specific cases. The medication could be injected locally using a laparoscopic or transvaginal approach. Agents like methotrexate, potassium chloride, and hyperosmolar glucose could be used in nonviable pregnancies. The use of MTX in the presence of a viable pregnancy is contraindicated, as it can lead to miscarriage or congenital malformations. Instead, sonographically guided local injection of KCL into the heart of the ectopic fetus can induce cardiac asystole. Hyperosmolar glucose may also be injected into the gestational sac, causing local dehydration, necrosis, and resolution of the trophoblastic tissue. Cornual and interstitial pregnancies can be managed by surgical excision or ultrasound-guided aspiration with KCL injection. Cervical heterotopic pregnancy may be treated through ultrasound-guided aspiration with cervical sutures, intra-cardiac KCL injection, vacuum aspiration following ligation of the descending cervical branches of the uterine arteries, electrodesiccation, or hysteroscopic resection. For ovarian ectopic pregnancy, laparoscopic wedge resection or ipsilateral oophorectomy is the preferred treatment [25, 67]. In cases of single-twin fetal demise, chorionicity, and gestational age at the time of demise are the key factors influencing the survival of the second twin. The perinatal survival rate is reported to be 83% for monochorionic twins and 100% for dichorionic twins [68,69].

Pregnancies achieved after ART are considered “high-risk” even when single, but especially when twin intrauterine gestation is present. In cases where there is disruption of the corpus luteum cyst up to 12 weeks after ectopic excision, irrespective of the number of embryos, progesterone support is strongly indicated [2]. In twins, the administration of additional progesterone, especially for the cervix, is under debate, but early cervical assessment is still considered crucial.

There has also been a lot of discussion concerning the role of cervical cerclage and vaginal progesterone in the treatment of cervical incompetence. From the author’s experience, the pregnant woman admitted had a history of cervical incompetence with unfavorable preterm birth at 22 weeks. After counseling, she was treated with combined therapy, cervical cerclage at 13 weeks, and vaginal progesterone 200 mg once a day. Conclusively, this treatment strategy ended in a favorable outcome [34]. In a systematic review, Conde-Agudelo et al. [70] stated that in singleton pregnancies with a short cervix in the second trimester and previous spontaneous preterm birth, either vaginal progesterone or cerclage are equally efficacious. On the contrary, Wang et al. noticed that cerclage is more beneficial than progesterone [71]. The same author mentioned that the effectiveness is similar when vaginal progesterone is added to the cervical cerclage. In another article, cerclage, vaginal progesterone, and cervical pessary appear to have similar effectiveness, while the use of single or combined therapy is still under discussion [72]. The results of twin gestations are also controversial. A recent review declared that vaginal progesterone decreases preterm birth and neonatal morbidity and mortality in women with a short cervix, while in another article, cervical pessary, progesterone, and cerclage do not show a significant effect in reducing the rate of preterm birth or perinatal morbidity in twins [73,74].

This systematic review has several limitations. First, the data are derived exclusively from case reports and series, which are inherently subject to reporting biases and lack comparative analyses. Second, the heterogeneity of the included cases, such as variations in ART protocols, patient demographics, and clinical management strategies, limits the generalizability of findings. Additionally, incomplete or missing data in some reports, particularly regarding long-term maternal and neonatal outcomes, hinder comprehensive analysis. Diagnostic approaches and management strategies varied significantly across cases, reflecting differences in clinician expertise and available resources. Finally, the lack of a standardized risk of bias assessment for case reports further complicates the evaluation of study quality and reliability. These limitations highlight the need for more robust, standardized reporting and prospective studies to better understand and manage heterotopic triplet pregnancies associated with ART.

## Conclusions

The term “heterotopic triplet pregnancy,” describing the presence of three embryos with one or two being ectopic, requires further establishment in the literature, particularly in the context of ART. In some cases, incomplete data recording highlights the need for systematic documentation in future occurrences of HTP. Pregnancy outcomes appear to be independent of ectopic rupture or hemoperitoneum, provided timely presentation and referral to appropriate healthcare facilities occur. Early diagnosis remains critical to avoiding complications, such as blood transfusion, and facilitating minimally invasive treatments like laparoscopy. This can only be achieved if clinicians are fully aware of this increasingly frequent condition. In cases of delayed or missed diagnosis, laparotomy remains the treatment of choice. It is important to note that OHSS can contribute to diagnostic challenges.

The well-documented recommendation to transfer only one or a maximum of two fertilized embryos in a single ovarian-controlled cycle must be emphasized to minimize the risks associated with multiple pregnancies, including heterotopic triplet pregnancies. A thorough clinical history, particularly in cases of known or suspected tubal pathology, is vital to early identification and management.

Given the increasing prevalence of heterotopic pregnancies with ART use, the authors recommend a comprehensive examination of the cervix and adnexa during every early vaginal ultrasound, even when intrauterine gestation is confirmed. Serial vaginal scans should also be considered in pregnancies induced by ART, conducted every one to two weeks, starting from the fifth week and continuing through the 10th-11th weeks of gestation. Obstetrical complication management strategies-such as addressing OHSS, cervical incompetence, demise of a twin, or premature rupture of membranes-should balance established protocols, clinician expertise, and patient preferences to optimize outcomes

## Appendices

The Risk of Bias Assessment is illustrated in Appendix A 

**Table 3:** Risk of bias assessment.

Study	Study design	Demographics reporting	Assisted reproductive technique intervention reporting	Outcome reporting	Selection bias	Reporting bias	Overall risk of bias
Payne S et al. [43]	Case Report	Yes	Yes	Yes	Low	Low	Low
Walker TA et al. [44]	Case Report	Yes	Yes	Yes	Low	Low	Low
Sondheimer SJ et al. [35]	Case Report	Yes	Yes	Yes	Low	Low	Low
Yovich JL et al. [3]	Case Report	Yes	Yes	Yes	Low	Low	Low
Porter R et al. [4]	Case Report	Yes	Yes	Yes	Low	Low	Low
Hanf V et al. [10]	Case Report	Yes	Yes	Yes	Low	Low	Low
Goldman JA et al. [ 5]	Case Report	Yes	Yes	Yes	Low	Low	Low
Chen SU et al. [11]	Case Report	Yes	Yes	Yes	Low	Low	Low
Smith S et al. [12]	Case Report	Yes	Yes	Yes	Low	Low	Low
Bassil S et al. [28]	Case Report	Yes	Yes	Yes	Low	Low	Low
Berliner I et al. [6]	Case Report	Yes	Yes	Yes	Low	Low	Low
Inion I et al. [7]	Case Report	Yes	Yes	Yes	Low	Low	Low
Barnett A et al. [13]	Case Report	Yes	Yes	Yes	Low	Low	Low
Nair P et al. [8]	Case Report	Yes	Yes	Yes	Low	Low	Low
Ludwig M et al. [9]	Case Report	Yes	Yes	Yes	Low	Low	Low
Oliveira FG et al. [29]	Case Report	Yes	Yes	Yes	Low	Low	Low
Pan HS et al. [36]	Case Report	Yes	Yes	Yes	Low	Low	Low
Nikolaou DS et al. [14]	Case Report	Yes	Yes	Yes	Low	Low	Low
Hoopmann M. [30]	Case Report	Yes	Yes	Yes	Low	Low	Low
Sayin NC et al. [45]	Case Report	Yes	Yes	Yes	Low	Low	Low
Strelec M et al. [15]	Case Report	Yes	Yes	Yes	Low	Low	Low
Bhat SM et al. [16]	Case Report	Yes	Yes	Yes	Low	Low	Low
Childs AJ et al. [38]	Case Report	Yes	Yes	Yes	Low	Low	Low
Cirillo D et al. [39]	Case Report	Yes	Yes	Yes	Low	Low	Low
Muller Vranjes A et al. [46]	Case Report	Yes	Yes	Yes	Low	Low	Low
Dundar O et al [31]	Case Report	Yes	Yes	Yes	Low	Low	Low
Gupta A et al [37]	Case Report	Yes	Yes	Yes	Low	Low	Low
Divry V et al. [17]	Case Report	Yes	Yes	Yes	Low	Low	Low
Berkes E et al. [18]	Case Report	Yes	Yes	Yes	Low	Low	Low
Kasum M et al. [19]	Case Report	Yes	Yes	Yes	Low	Low	Low
Bugatto F et al. [2]	Case Report	Yes	Yes	Yes	Low	Low	Low
Timor-Tritsch I et al [20]	Case Report	Yes	Yes	Yes	Low	Low	Low
Tomic V et al. [21]	Case Report	Yes	Yes	Yes	Low	Low	Low
Litwicka K et al. [32]	Case Report	Yes	Yes	Yes	Low	Low	Low
Okamura Y et al. [22]	Case Report	Yes	Yes	Yes	Low	Low	Low
Bornstein E et al. [23]	Case Report	Yes	Yes	Yes	Low	Low	Low
Gozukucuk M et al [24]	Case Report	Yes	Yes	Yes	Low	Low	Low
Osmanagaoglou M et al [40]	Case Report	Yes	Yes	Yes	Low	Low	Low
Herrera E et al [33]	Case Report	Yes	Yes	Yes	Low	Low	Low
Lin CK et al. [25]	Case Report	Yes	Yes	Yes	Low	Low	Low
Delrieu et al [26]	Case Report	Yes	Yes	Yes	Low	Low	Low
Fukuda T et al. [47]	Case Report	Yes	Yes	Yes	Low	Low	Low
Felekis T et al. [1]	Case Report	Yes	Yes	Yes	Low	Low	Low
Buca DI et al. [48]	Case Report	Yes	Yes	Yes	Low	Low	Low
Kasap B et al [49]	Case Report	Yes	Yes	Yes	Low	Low	Low
Nitke S et al. [50]	Case Report	Yes	Yes	Yes	Low	Low	Low
Hsieh BC et al. [51]	Case Report	Yes	Yes	Yes	Low	Low	Low
Gaudier FL et al. [52]	Case Report	Yes	Yes	Yes	Low	Low	Low
Al Mulla A et al [53]	Case Report	Yes	Yes	Yes	Low	Low	Low
Vitner D et al. [54]	Case Report	Yes	Yes	Yes	Low	Low	Low
Mustafa KB et al. [55]	Case Report	Yes	Yes	Yes	Low	Low	Low
Jonler M et al. [56]	Case Report	Yes	Yes	Yes	Low	Low	Low
Riestenberg CK et al. [57]	Case Report	Yes	Yes	Yes	Low	Low	Low
Korkontzelos I et al. [34]	Case Report	Yes	Yes	Yes	Low	Low	Low
Bhattacharya R et al [41]	Case Report	Yes	Yes	Yes	Low	Low	Low
Morong J et al.423]	Case Report	Yes	Yes	Yes	Low	Low	Low
Bhoi NR et al [27]	Case Report	Yes	Yes	Yes	Low	Low	Low
